# Pan-Cancer Analysis of TCGA Data Revealed Promising Reference Genes for qPCR Normalization

**DOI:** 10.3389/fgene.2019.00097

**Published:** 2019-03-01

**Authors:** George S. Krasnov, Anna V. Kudryavtseva, Anastasiya V. Snezhkina, Valentina A. Lakunina, Artemy D. Beniaminov, Nataliya V. Melnikova, Alexey A. Dmitriev

**Affiliations:** Engelhardt Institute of Molecular Biology, Russian Academy of Sciences, Moscow, Russia

**Keywords:** cancer, gene expression, reference genes, quantitative PCR, data normalization, RNA-Seq, TCGA, CrossHub

## Abstract

Quantitative PCR (qPCR) remains the most widely used technique for gene expression evaluation. Obtaining reliable data using this method requires reference genes (RGs) with stable mRNA level under experimental conditions. This issue is especially crucial in cancer studies because each tumor has a unique molecular portrait. The Cancer Genome Atlas (TCGA) project provides RNA-Seq data for thousands of samples corresponding to dozens of cancers and presents the basis for assessment of the suitability of genes as reference ones for qPCR data normalization. Using TCGA RNA-Seq data and previously developed CrossHub tool, we evaluated mRNA level of 32 traditionally used RGs in 12 cancer types, including those of lung, breast, prostate, kidney, and colon. We developed an 11-component scoring system for the assessment of gene expression stability. Among the 32 genes, *PUM1* was one of the most stably expressed in the majority of examined cancers, whereas *GAPDH*, which is widely used as a RG, showed significant mRNA level alterations in more than a half of cases. For each of 12 cancer types, we suggested a pair of genes that are the most suitable for use as reference ones. These genes are characterized by high expression stability and absence of correlation between their mRNA levels. Next, the scoring system was expanded with several features of a gene: mutation rate, number of transcript isoforms and pseudogenes, participation in cancer-related processes on the basis of Gene Ontology, and mentions in PubMed-indexed articles. All the genes covered by RNA-Seq data in TCGA were analyzed using the expanded scoring system that allowed us to reveal novel promising RGs for each examined cancer type and identify several “universal” pan-cancer RG candidates, including *SF3A1, CIAO1*, and *SFRS4*. The choice of RGs is the basis for precise gene expression evaluation by qPCR. Here, we suggested optimal pairs of traditionally used RGs for 12 cancer types and identified novel promising RGs that demonstrate high expression stability and other features of reliable and convenient RGs (high expression level, low mutation rate, non-involvement in cancer-related processes, single transcript isoform, and absence of pseudogenes).

## Introduction

Quantitative PCR (qPCR) is the most widely used technique for quantification of gene expression. qPCR is rapid, has a very high dynamic range of mRNA level quantification and provides a measurement of even small gene expression alterations in a large number of samples. The most common and convenient approach for qPCR data normalization assumes mRNA quantification of a reference gene (RG) with stable expression level between the samples under study (Huggett et al., 2005). It is a bottleneck of qPCR, and the reliability of qPCR results strongly depends on the selection of appropriate RGs. This issue becomes more acute when it comes to assessing the moderate changes in the mRNA level of target genes (<2-fold).

The problem of selecting appropriate RGs is especially crucial in cancer studies because of the presence of several molecular subtypes within a histological type and, moreover, a unique molecular portrait of each tumor (Janssens et al., 2004). Despite the fact that almost 30 years have passed since the moment when the issue of picking appropriate RGs had arisen, there is still no consensus (Janssens et al., 2004; Rubie et al., 2005; Gur-Dedeoglu et al., 2009; Ibusuki et al., 2013; Zhao et al., 2018). Many studies indicate that most frequently used RGs (*GAPDH, ACTB, B2M, etc*.) have a wide but limited field of applicability: they should not be illegibly used for a wide spectrum of diseases or stress conditions (Barber et al., 2005; Rubie et al., 2005; Kozera and Rapacz, 2013; Chapman and Waldenstrom, 2015). To increase the reliability of qPCR data, one should use at least two or more RGs that are not co-expressed with each other (Chapman and Waldenstrom, 2015). The most rigorous approach is to analyze a panel of 5–20 RGs and choose those with the most stable expression for a current study. Several tools have been developed for these purposes: geNorm (Vandesompele et al., 2002), NormFinder (Andersen et al., 2004), BestKeeper (Pfaffl et al., 2004). However, the vast part of researchers do not perform the analysis of RG suitability and just rely on the existing literature data concerning the object of study (Chapman and Waldenstrom, 2015).

Whole-transcriptomic data allow us to look at the problem from the other side. RNA-Seq opens up great opportunities for a complex expression analysis and identifying trends in the mRNA level changes of groups of genes between the samples. RNA-Seq data are free of bias that comes from the instability of RG expression. The most common RNA-Seq data normalization strategy is based on the assumption that the mRNA level of the majority of genes is stable. This method is implemented in popular RNA-Seq differential expression analysis packages, including edgeR [trimmed mean of M-values method, TMM; Robinson et al., 2010], DESeq2 (Love et al., 2014), and others. There are other normalization strategies: by total read count, by upper quartile or median values, FPKM/RPKM, TPM, “remove unwanted variation” (RUV) (Risso et al., 2014); as well as machine-learning approaches: RNA-Seq by Expectation-Maximization (RSEM) (Li and Dewey, 2011) and Sailfish (Patro et al., 2014). Despite the diversity of the methods, in most cases, they give rather similar results, which differ by 20–30%, with the exception of some cases when the expression of half or more of genes is changed significantly (Dillies et al., 2013; Li et al., 2015; Zyprych-Walczak et al., 2015; Evans et al., 2018).

Analysis of highly representative RNA-Seq and microarray datasets is very attractive in terms of the identifying stably expressed RGs for human (Popovici et al., 2009; Tilli et al., 2016; Chen et al., 2017; Chim et al., 2017; Hoang et al., 2017) or other organisms (Alexander et al., 2012; Carmona et al., 2017; Zhou et al., 2017). This approach is valuable for the detection of novel housekeeping gene candidates with constitutively stable mRNA level.

In 2016, Tilli et al. suggested a strategy including the large-scale screening of potential RGs from RNA-Seq data with further validation by qPCR and applied it for breast cancer (Tilli et al., 2016). The authors analyzed datasets of The Cancer Genome Atlas (TCGA) and Gene Expression Omnibus (GEO) and found that several non-traditional RGs, *CCSER2, SYMPK, ANKRD17*, as well as known RG *PUM1* demonstrated the least expression variability in breast cancer samples and normal tissues (Tilli et al., 2016). The similar approach was realized by Chen et al. for the identification of reference mRNA and miRNA suitable for human esophageal squamous cell carcinoma studies (Chen et al., 2017). It allowed authors to identify non-standard RG candidates—*DDX5, LAPTM4A, P4HB*, and *RHOA*.

TCGA is the largest resource in the field of cancer biology that is aimed at the discovery of the molecular features of various cancer types (https://cancergenome.nih.gov/). TCGA database includes genomic, transcriptomic, and epigenetic data for 33 human cancer types represented with more than 11,000 individual samples. In the present work, we analyzed TCGA transcriptome sequencing data in order to evaluate the expression stability of widely used RGs and identify novel RG candidates in 12 most common cancer types. The use of representative TCGA sample sets allows us to pay extra attention to the overall stability of mRNA level and presence of outliers, the cases of dramatic expression “blow up” or falling down in single samples. Besides the data on mRNA level, we took into account if this is a well-studied gene or not (by evaluating the number of mentions in PubMed-indexed titles/abstracts), if a gene is involved in cancer-associated biological processes like cell cycle, differentiation, and adhesion (using Gene Ontology). Additionally, we evaluated if a gene is highly mutated (using TCGA data on somatic mutations) that indicates its implication in cancer. Also, we tried to minimize the number of pseudogenes and alternatively spliced transcripts in order to improve usability: the presence of pseudogenes makes it difficult to pick up cDNA-specific primer pairs, and the presence of alternative transcripts complicates the expression analysis and may lead to flawed results. We integrated all the parameters listed above into a single scoring system. Finally, we looked for genes that demonstrate cross-tissue expression stability and may represent “universal” pan-cancer RGs.

## Materials and Methods

In the present work, we focused on TCGA data for 12 cancer types for which RNA-Seq data were available for representative sample sets: at least 100 tumor (T) and 20 normal (N) tissue samples. The data were processed with a modified version of CrossHub (Krasnov et al., 2016), a tool for the multi-way analysis of TCGA transcriptomic and genomic data. Read counts data were downloaded from the TCGA data portal (https://portal.gdc.cancer.gov/) and normalized using the TMM method and then recalculated for 1 million library size. The derived CPM (read counts per million) values were used as a measure of mRNA level of a gene for further expression stability analysis.

In order to assess gene expression stability, we developed a scoring system, which included several components (*S*_*i*_) responsible for T-N expression level difference, expression level stability within pools of N and T samples, and correlations of mRNA level with clinical and pathological characteristics [disease stage, TNM (tumor, node, metastasis) classification, follow-up status]. Each scoring component *S*_*i*_ takes values from 0 to 100. All *S*_*i*_ are taken with different weights (W_*i*_), which reflect the importance of component. Overall expression scoring *S*^Exp^ is calculated as follows:

$$\begin{array}{l} S^{\text{Exp}}={\left(\overset{N}{\underset{i=1}{\prod }}{\left(S_{i}+\text{C}{\text{A}}_{i}\right)}^{{\text{W}}_{i}}\right)}^{1/\sum_{i=1}^{N}W_{i}} \end{array}$$

where:

- CA_*i*_ is a constant summand, which is used to mitigate the impact of zero values of *S*_*i*_;
- W_*i*_ is weight of a component *S*_*i*_ (*i* = 1 … N);
- *N* is a number of components, *N* = 48.

Values of these parameters are presented in Table 1.

**Table 1:** Components of the scoring function.

**Component**	**Factor**	Variable (x = …)^*^	**IV**	**IP**	**CS**	**Sq**	**CA**	**W**	**Number of times applied**
**EXPRESSION SCORING**									
S_DP_	T-N expression level difference (pooled samples)	Abs (log_2_FC_P_) _10−90_	0.05	0.25	2.5	1	0	4	1 (all samples)
S_DL_	T-N expression level difference (paired samples)	Abs (Average(log_2_FC_L_)_10−90_)							1 (paired samples)
S_DoO_	T-N expression level difference: outliers, overexpression	Abs (Average(log_2_FC_L_)_90−100_)	0.1	0.7	2.5	1	10	1	1 (paired samples)
S_DoU_	T-N expression level difference: outliers, underexpression	Abs (Average(log_2_FC_L_)_0−10_)							1 (paired samples)
S_DLc_	Cumulative T-N expression difference among paired samples	Average (Abs(log_2_FC_L_)_10−90_)	0.1	0.5	2.5	1	5	2	1 (paired samples)
S_EStD_	Expression level stability: standard deviation	StDev (CPM)_10−90_/Average (CPM)_10−90_	0.1	0.3	2	1	5	1.5	2 (all samples: normal and tumor)
S_EoH_	Expression level stability: outliers (high expression)	log_2_ (Average(CPM)_90−100_/Average (CPM)_10−90_)	0.1	0.7	2.5	1	5	0.75	2 (all samples: normal and tumor)
S_EoL_	Expression level stability: outliers (low expression)	log_2_ (Average(CPM)_10−90_/Average (CPM)_0−10_)							2 (all samples: normal and tumor)
S_EA_	Average expression level	1/log_2_ (CPM)_10−90_	0.07	0.15	3	1	0	6	1 (all tumor samples)
S_Cp_	Correlations of expression with clinical parameters (*p*-values)	-log_2_ (*p*-value)	2	4	3	0.3	5	0.3	18 (3 × 6; 3: CPM_10−90_ all tumor samples, CPM_10−90_ all normal samples, (log_2_FC_L_)_10−90_; 6: pathologic TNM classification, pathologic stage, follow-up—person neoplasm cancer status, follow-up—treatment success)
S_Cr_	Correlations of expression with clinical parameters (*r*_s_)	Abs (*r*_s_)	0.1	0.25	2.5	0.3	5	0.2	18 (the same as above)
**“ANTI-SCORINGS”**									
S^Mut^	Percentile of mutation rate		75	95	4	1			
S^Isoforms^	Number of transcript isoforms		1	3	2	0.4			
S^Pseudogenes^	Number of pseudogenes		0	2	2	0.4			

* *Percentiles, which were taken into calculation, are indicated as a subscript*.

*IV, ideal value; IP, inflection point; CS, curve slope; Sq, “squeeze”; CA, constant add; W, weight; Abs (…), absolute value; Average (…), mean value; CPM, counts per million, gene expression level; FC_P_, ratio of the average CPM in a pool of tumor samples to the average CPM in a pool of normal samples; FC_L_, ratio of CPM values between tumor and matched normal tissue (per each paired sample); StDev (…), standard deviation; r_s_, Spearman's correlation coefficient*.

Each individual component *S*_*i*_ is calculated with a common parametric formula:

$$\begin{array}{l} S_{i}\;=\frac{100}{1+\text{Sq}\times {\left(\frac{\text{max}\left(x-\text{IV};0\right)}{\text{IP}-\text{IV}}\right)}^{\text{CS}}} \end{array}$$

This formula provides a (1-sigma)-like function with a customizable inflection point, tilt, and region of maximal score values. The function takes values from 0 to 100. Here:

- *x* is a variable to be scored (see Table 1).
- IV is an “ideal value.” All cases with *x* ≤ IV would produce the maximum score (100). For example, *S*_DP_, the component responsible for T-N expression level fold change (see Table 1) would be equal to 100 for any log_2_FC_P_ between −0.05 and +0.05 since IV = 0.05.
- IP is an “inflection point.” In this point, there is the maximum decrease rate of *S*_*i*_. When *x* is equal to IP and Sq = 1 (Sq takes these value for most *S*_*i*_), the scoring component *S*_*i*_ = 50. Ideally, IP value should reflect the marginally acceptable value of *x*. For example, the relative standard deviation of gene expression (RelSD) values from 0 to 0.25 are appropriate, but RelSD = 0.4 … 0.5 is almost unacceptable. For the corresponding component (S_EStD_), we chose IV = 0.1 and IP = 0.3 (see Table 1).
- CS is a “curve slope.” The greater CS value, the stronger *S*_*i*_ decrease rate. Higher CS values should be assigned to more important scoring components.
- Sq is a “Squeeze,” this is an auxiliary parameter. For most scoring components, it is equal to 1.

**Figure 1:**
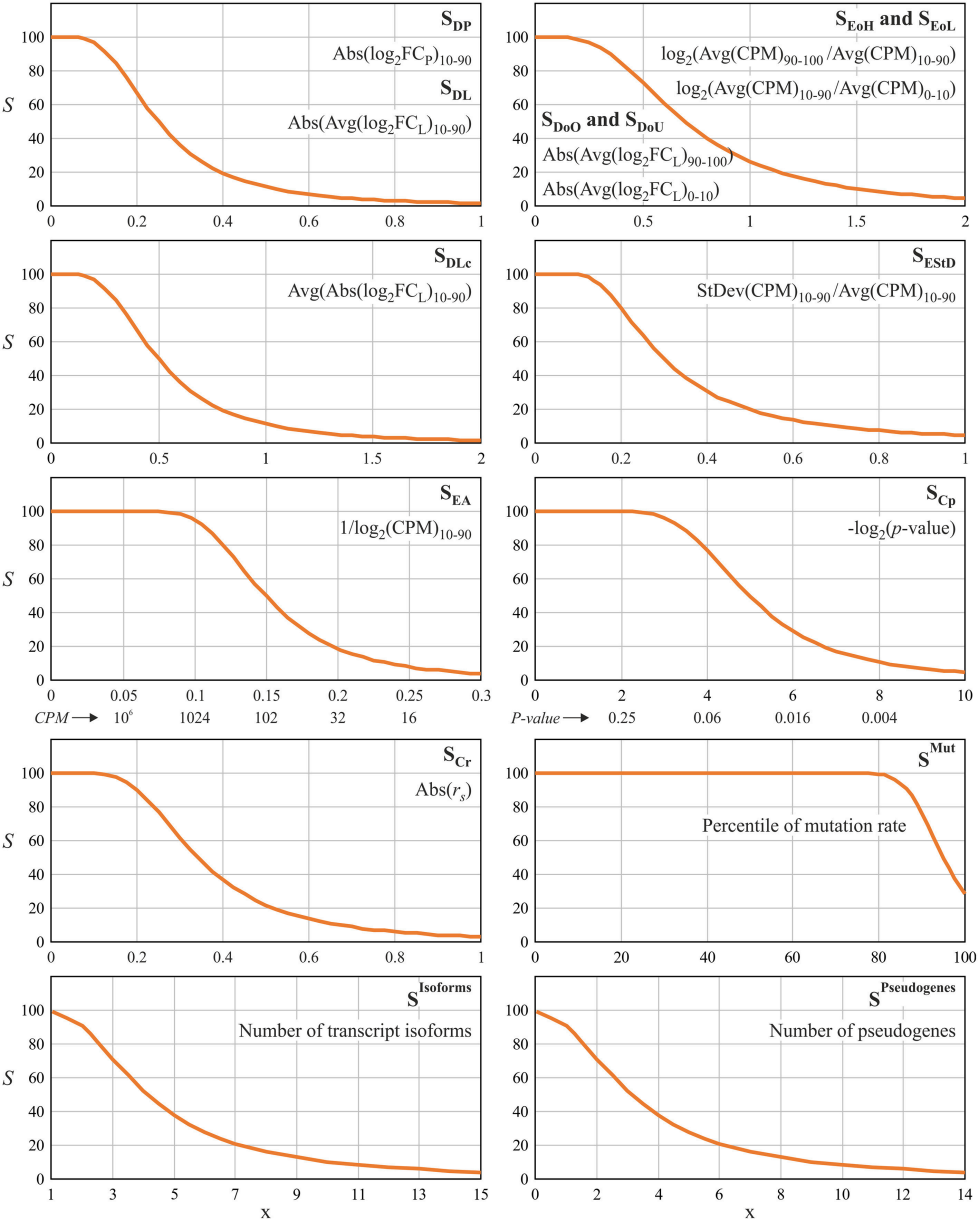
Scoring functions used for evaluation of gene suitability for qPCR data normalization. Percentiles, which were taken into calculation, are indicated as a subscript. Abs(…), absolute value; Avg(…), mean value; CPM, counts per million, gene expression level; FC_P_, ratio of the average CPM in a pool of tumor samples to the average CPM in a pool of normal samples; FC_L_, ratio of CPM values between tumor and matched normal tissue (per each paired sample); StDev(…), standard deviation; r_s_, Spearman's correlation coefficient.

All scoring components *S*_*i*_ and parameters (IV, IP, CS, Sq) are presented in Table 1. The derived scoring functions are shown in Figure 1.

Two components, S_DP_ and S_DL_, are responsible for T-N expression level difference. This is the major factor of RG suitability. S_DP_ is calculated for pooled, and S_DL_–for paired samples. Hence, we applied the strongest scoring parameters (IV = 0.05, IP = 0.25, CS = 2.5) and assigned high weight (W = 4) for these two components. S_DP_ (or S_DL_) would be equal to 50 if the absolute value of average log_2_FC_P_ (or log_2_FC_L_) is equal to IP = 0.25, i.e., fold change between tumor and normal is about 20%. We chose IV = 0.05–0.1 for all the components that are responsible for expression level (S_DP_, S_DL_, S_DoO_, S_DoU_, S_DLc_, S_EStD_, S_EoH_, S_EoL_). This means that 5–10% mRNA level changes are ignored.

S_DP_ and S_DL_ are calculated using the trimmed means of either CPM (pooled sample) or log_2_FC_L_ (paired samples). Only values from 10 to 90th percentiles are included. To take into account T-N expression outliers, we added two other scorings, S_DoO_ and S_DoU_, that are responsible for the upper and lower deciles of log_2_FC_L_. For these components, we assigned increased IP value (IP = 0.7) since it is expected that Abs[Average(log_2_FC_L_)_90−100_] calculated for 90–100th percentiles of log_2_FC_L_ will be much greater than such value calculated for 10–90th percentiles.

S_EStD_, S_EoH_, S_EoL_ are responsible for evaluating expression stability within pools of normal and tumor samples. S_EStD_ scores trimmed standard deviation of CPM values (10–90th percentiles), and S_EoH_ (or S_EoL_) is responsible for outliers with high (or low) mRNA level (in terms of CPM). Additionally, we included scoring for average expression level (S_EA_) and set high weight (*W* = 6) for this component in order to completely exclude genes with low mRNA level from the analysis.

Finally, we added scorings for correlations between gene expression and six clinical and pathological characteristics: pathologic TNM classification (separately for T, N, and M indexes), pathologic stage, follow-up person neoplasm cancer status and follow-up treatment success status. S_Cr_ is the component responsible for Spearman's correlation coefficient, and S_Cp_–for correlation *p*-value. IV values were chosen in such a way that cases with *p* > 0.25 and |*r*_s_| < 0.1 have score equals to 100. In total, each of these two components is taken 18 times: 6 clinical characteristics are analyzed for associations with CPM in tumor samples, CPM in normal samples and T-N expression fold change (paired samples). Hence, we assigned low weights—*W* = 0.2 and 0.3 for S_Cr_ and S_Cp_, respectively.

Besides stable and high enough expression level, an appropriate RG should also demonstrate a low mutation rate, single transcript isoform, and absence of pseudogenes in order to avoid problems with PCR priming and ensure the rigorous evaluation of mRNA level. The mutation rate of a gene was assessed using TCGA data on somatic mutations. The number of transcript isoforms (per gene) was obtained from the Ensembl human genome annotation (hg38, release 88). The number of pseudogenes (per gene) was derived from psiCube (Sisu et al., 2014). Therefore, we extended the scoring system with three additional components, “anti-scorings” (Table 1 and Figure 1). The resulting score *S*^Final^ is calculated as follows:

$$\begin{array}{l} S^{\text{Final}}=S^{\text{Exp}}\cdot S^{\text{Mut}}\cdot S^{\text{Isoforms}}\cdot S^{\text{Pseudogenes}} \end{array}$$

Next, we tried to find RGs that are stably expressed across multiple tissues and cancer types. For this purpose, we calculated the pan-cancer score as follows:

$$\begin{array}{l} S_{\text{Pan}-\text{cancer}}^{\text{Final}}=S_{\text{Pan}-\text{cancer}}^{\text{Exp}\&\text{Mut}}\cdot S^{\text{Isoforms}}\cdot S^{\text{Pseudogenes}} \end{array}$$

where:

$$\begin{array}{l} S_{\text{Pan}-\text{cancer}}^{\text{Exp}\&\text{Mut}}={\left(\frac{\sum_{j=1}^{M}{\left(S_{j}^{\text{Exp}}\cdot S_{j}^{\text{Mut}}+\text{CA}\right)}^{k}}{M}\right)}^{1/k} \end{array}$$

where *M* = 12 (a number of cancer types analyzed); *k* = −0.4 (negative *k* value implies that the pan-cancer score is a harmonic mean of individual scores); *CA* = 12 (a constant add).

Finally, we assessed the involvement of a gene in cancer-related processes on the basis of Gene Ontology (GO; The Gene Ontology, 2017) data and mentions in the articles indexed by PubMed (titles and abstracts).

A RG should not be involved in cellular processes that are frequently altered in cancer. A penalty system based on GO data was developed. We evaluated the involvement of a gene in 6 cancer-associated biological processes: cell cycle, differentiation, stress response, immune response, angiogenesis, adhesion, and cell communication. The relation of a gene to each of these processes was followed by the assignment of penalty points (from 2 to 5). Finally, these points were summed up. According to this system, a gene is penalized (1) with 5 points if its GO annotation contains at least one keyword related to cell cycle process: *cell cycle, cell division, cell growth, cell proliferation, apoptosis, apoptotic process, cell death, MAPK cascade, tumor, oncogenic, apoptotic*; (2) with 4 points if GO annotation contains a keyword related to cell differentiation: *cell differentiation, epithelial to mesenchymal transition, mesenchymal to epithelial transition, stem cell, fetal, embryonic, embryonal, embryo, gastrulation, tissue development, cellular developmental process, organ development*; (3) with 3 points for stress response related processes: *response to stress, DNA damage, DNA repair*; (4) with 2 points for inflammation and immune response: *inflammation, inflammatory, immune response, T cell activation, macrophage activation, antigen*; (5) with 2 points for angiogenesis: *angiogenesis*; (6) with 2 points for intercellular interactions: *cell communication, cell-cell signaling, cell adhesion, cell motility, cell migration*. Thus, a gene may have a maximum of 5 + 4 + 3 + 2 + 2 + 2 = 18 penalty points.

The more accurately the gene is annotated, the more likely it is to find one of the keywords in its annotation. Therefore, GO penalty should be normalized taking into account the number of assigned GO terms for the gene. On the other hand, the better the gene is annotated, the more extensively it is studied, and such genes represent more attractive candidates. In order to keep a balance between these two factors, we introduced normalization coefficient evaluated as the total number of GO terms (assigned for the gene) to the power of 0.3. If a gene lacked sufficient GO annotation (<3 GO terms), we assigned it 10 penalty points.

The number of PubMed-indexed articles with the mention of a gene name or its aliases was evaluated to assesses how well a gene is studied. Next, within this pool of gene-related publications, the number of cancer-related articles was also evaluated. One of the following words should be present in an article title to be treated as cancer-related: *cancer, tumor*, ^*^carcinoma, sarcoma, glioma, glioblastoma, and other keywords.

The described components (GO and Pubmed) were not included in the main scoring and were only used for manual exclusion of cancer-associated genes. Besides, functional annotations from RefSeqGene (https://www.ncbi.nlm.nih.gov/refseq/rsg/) were added to each gene.

When revealing optimal RG pairs for each of examined cancer types, we paid special attention to the co-expression of RG candidates to avoid genes with a pronounced correlation between their mRNA levels. To implement the scoring system, we modified our previously developed CrossHub tool (the updated version can be downloaded at https://sourceforge.net/projects/crosshub/).

## Results

We performed the analysis of 12 cancer types from the TCGA project that have RNA-Seq data for representative sample sets: 285-1095 tumor and 19-113 matched normal tissues. These are: breast invasive carcinoma (BRCA), lung adenocarcinoma (LUAD), lung squamous cell carcinoma (LUSC), kidney renal cell carcinoma (KIRC), kidney renal papillary cell carcinoma (KIRP), prostate adenocarcinoma (PRAD), colon adenocarcinoma (COAD), head and neck squamous cell carcinoma (HNSC), liver hepatocellular carcinoma (LIHC), stomach adenocarcinoma (STAD), thyroid carcinoma (THCA), and bladder urothelial carcinoma (BLCA). For the remaining TCGA cancer types, RNA-Seq data were available only for a few normal tissue samples, and this makes it impossible to use such datasets for the discovery of reliable RGs.

First, we assessed the expression stability of a set of 32 frequently used RGs in 12 selected cancer types: *ACTB, ALAS1, B2M, CDKN1A, G6PD, GAPDH, GUSB, HBB, HMBS, HPRT1, HSP90AB1, IPO8, LDHA, NONO, PGK1, POP4, PPIA, PPIH, PSMC4, PUM1, RPL13A, RPL30, RPLP0, RPS17, RPS18, SDHA, TBP, TFRC, UBC, YWHAZ, TUBB, RPN1*. This set of 32 RGs was composed of commercially available RG panels: Roche “Human Reference Gene Panel, 384” (Switzerland), TATAA “Reference Gene Panel Human” (Sweden), and Bio-Rad “Reference Genes H384” (USA). In total, 31 unique genes are included in the panels, plus we added the *RPN1* gene, which was identified by us earlier as a reliable RG for lung, kidney, and colorectal cancers (Krasnov et al., 2011; Fedorova et al., 2015). Expression stability scores were calculated for each gene in each examined cancer type. The results for the top 5 genes are presented in Table 2 and full data—in Supplementary Table 1. In almost each cancer type, there were 1–10 genes with expression score about 70 or more (with a theoretical maximum of 100), which can be considered as moderately high score value. PRAD and THCA demonstrated the highest number of genes with stable mRNA level-10 and 7, respectively. Only in BCLA, all the genes had scores below 70, possibly because of potential bias due to a small number of matched normal tissues (19—the smallest number among the cancer types examined). The cross-tissue analysis of 12 cancer types revealed that the most stably expressed genes were: *PUM1* (*S*^Exp^ = 70), *IPO8* (*S*^Exp^ = 61), *UBC* (*S*^Exp^ = 60), *ACTB* (*S*^Exp^ = 55), and *RPN1* (*S*^Exp^ = 54). *GAPDH*, one of the most frequently used RGs, showed one of the least stability of mRNA level—position 25 out of 32 (*S*^Exp^ = 32). According to the obtained results, *GAPDH* can be reasonably applied as a RG only in prostate and stomach adenocarcinomas. *RPN1* gene suggested by us demonstrated high expression stability score in lung, renal, colon, liver, thyroid, and prostate cancers.

**Table 2:** Top 5 traditionally used reference genes with the highest expression scores in 12 cancer types.

**Cancer type**	**1**		**2**		**3**		**4**		**5**	
	**Gene**	***S*^Exp^**	**Gene**	***S*^Exp^**	**Gene**	***S*^Exp^**	**Gene**	***S*^Exp^**	**Gene**	***S*^Exp^**
BRCA	***UBC***	82.1	***PUM1***	75.7	*IPO8*	71.8	*RPLP0*	69.8	*RPS18*	66.2
LUAD	***UBC***	79.8	*ACTB*	76.4	***PUM1***	69.6	*RPN1*	67.9	*RPL13A*	65.5
LUSC	***UBC***	81.4	*IPO8*	72.9	*ACTB*	71.4	***PUM1***	70.7	*RPL13A*	66.3
KIRC	***NONO***	82.6	*HSP90AB1*	73.2	***RPN1***	69.7	*YWHAZ*	68.7	*PSMC4*	64.7
KIRP	***PUM1***	70.3	***PSMC4***	66.0	*PGK1*	63.2	*ALAS1*	61.7	*IPO8*	61.1
PRAD	***SDHA***	80.8	*YWHAZ*	78.4	*PSMC4*	76.2	***PUM1***	76.1	*UBC*	75.8
COAD	***PUM1***	76.9	*GUSB*	73.4	***UBC***	72.8	*ACTB*	72.0	*IPO8*	71.6
HNSC	***RPL30***	73.4	***PUM1***	72.7	*IPO8*	68.1	*ACTB*	64.2	*PSMC4*	63.1
LIHC	***RPN1***	82.3	***ACTB***	80.9	*UBC*	78.4	*PUM1*	65.7	*RPS17*	56.4
STAD	***IPO8***	71.7	***RPL30***	71.0	*GAPDH*	69.7	*RPLP0*	68.7	*PUM1*	68.1
THCA	***RPN1***	84.4	*HSP90AB1*	84.3	***PUM1***	80.0	*TUBB*	79.2	*YWHAZ*	76.0
BLCA	***SDHA***	66.3	***PUM1***	65.9	*HSP90AB1*	63.3	*RPL30*	62.2	*RPS17*	61.2
Cross-tissue	*PUM1*	70.1	*IPO8*	60.8	*UBC*	59.8	*ACTB*	54.7	*RPN1*	54.3

*Optimal pairs of reference genes for each cancer type are shown in bold*.

Next, for each of 12 cancer types, we searched for a pair of the most suitable RGs focusing on *S*^Exp^ values and correlation between mRNA levels of genes in a pair. As a result, we revealed 12 optimal pairs of RGs with *S*^Exp^ above 65 for each gene and absence of co-expression (Table 2 and Supplementary Table 1). *PUM1* came into the pair of RGs for 9 out of 12 cancer types.

It should be noted that genes with high *S*^Exp^ values may be inconvenient in practice because of the presence of numerous pseudogenes, alternatively spliced transcripts or a high mutation rate. Among the traditionally used RGs with high expression scores, only 3 genes met the requirements—*PUM1, IPO8*, and *RPN1*. These genes have no pseudogenes, one (*RPN1*), or two (*PUM1* and *IPO8*) transcript isoforms, and relatively low mutation rate in examined cancer types.

**Figure 2:**
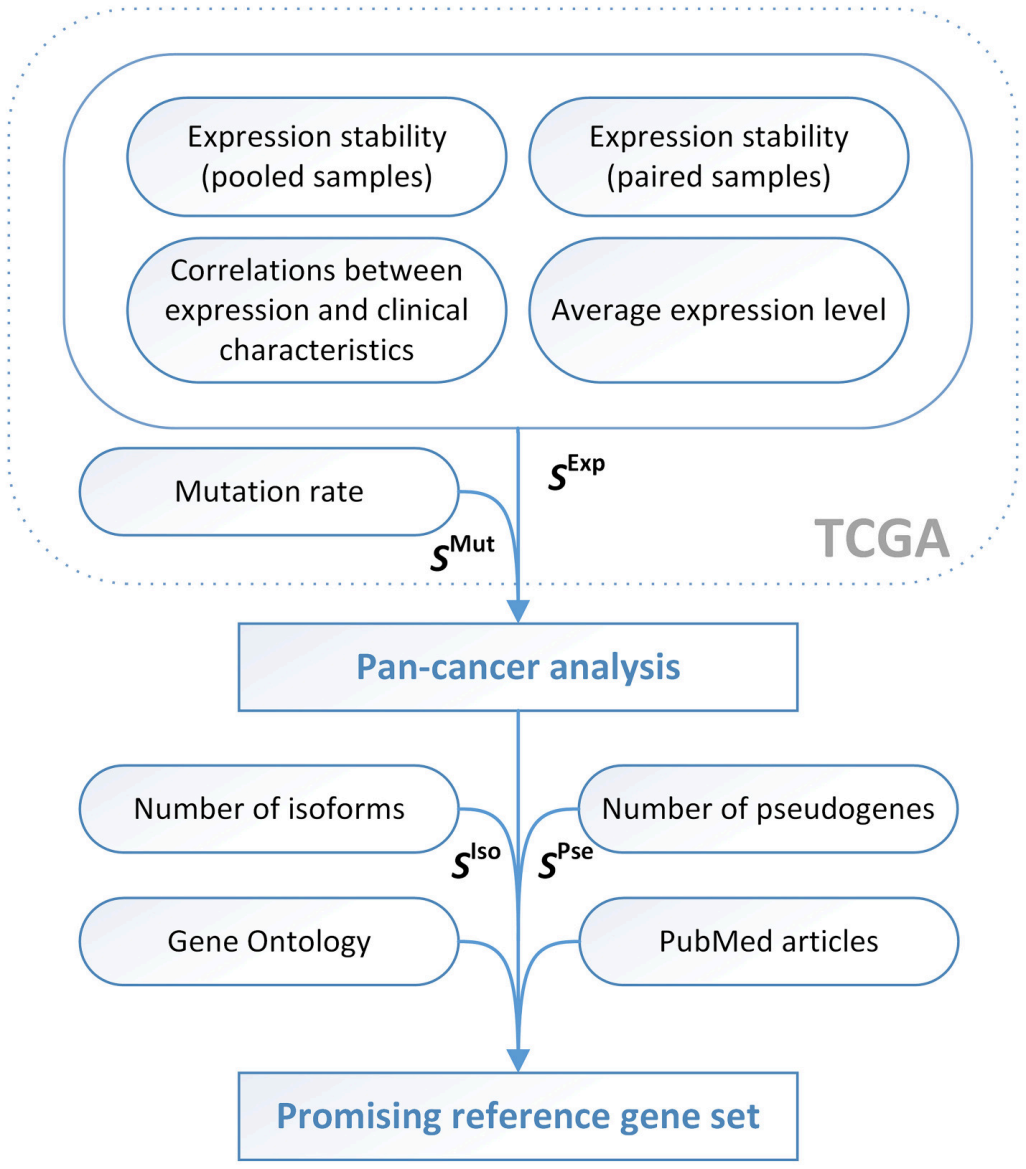
The pipeline for identification of promising reference genes for qPCR data normalization in cancer studies. *S*^Iso^ − *S*^Isoforms^, *S*^Pse^ − *S*^Pseudogenes^.

Using the expanded scoring system (Figure 2), in which 3 “anti-scorings” counting mutation rate, number of transcript isoforms and pseudogenes were included, we analyzed a complete list of human genes in order to reveal the most prominent pan-cancer RG candidates (Supplementary Table 2). Top 10 pan-cancer RG candidates included *MBTPS1, HNRNPA0, SF3A1, SF3B2, GGNBP2, HNRNPUL2, SFRS3, RTF1, CIAO1, TM9SF3*. All these genes had stable and high enough mRNA level and low mutation rate in most of 12 cancer types, only one annotated transcript isoform and no pseudogenes. Taking into account PubMed article search, GO annotations, and RefSeqGene information, we selected three most promising RG candidates—*SF3A1, CIAO1*, and *SFRS4*.

## Discussion

The use of inappropriate RGs leads to unreliable data and nullifies potentially high accuracy of a qPCR technique in the evaluation of differential gene expression. The search for a RG with a stable mRNA level under experimental conditions represents a separate object of research and is rarely performed during the original studies. RNA-Seq data of TCGA project offer a great opportunity for evaluating gene expression stability. Using our CrossHub tool, we developed a complex scoring system that allowed us to assess the suitability of 32 traditionally used RGs for qPCR data normalization in 12 cancer types characterized by high morbidity and mortality rates. The alterations of mRNA level were shown for a number of these genes, including the most frequently used *GAPDH*, in examined cancer types. The analysis across 12 cancer types revealed that *PUM1* and *IPO8* genes demonstrate the most stable expression among the 32 genes.

PUM1 (Pumilio RNA Binding Family Member 1) serves as a translational regulator of specific mRNAs by binding to their 3'-UTRs. It may be involved in translational regulation of embryogenesis, cell development, and differentiation. There are several functions that call into question its applicability as a RG. After growth factor stimulation, PUM1 binds to 3'-UTR of *CDKN1B/p27* tumor suppressor, inhibits its expression and promotes a rapid entry to the cell cycle (Kedde et al., 2010). PUM1 is capable of repressing many mitotic, DNA repair, and DNA replication factors (Lee et al., 2016). Moreover, some authors reported that *PUM1* promotes ovarian cancer proliferation, migration, and invasion (Guan et al., 2018). However, *PUM1* is identified as one of the most stably expressed genes in uterine cervical cancer (Tan et al., 2017), endometrial carcinoma (Ayakannu et al., 2015), gallbladder (Yu et al., 2015), leiomyoma (Almeida et al., 2014), breast (Ibusuki et al., 2013; Kilic et al., 2014), and non-small cell lung (Soes et al., 2013) cancers. This gene has only 2 transcript isoforms and no pseudogenes that makes it even more attractive for use as a reference one.

Recently, Tilli et al. performed a screening of breast cancer RNA-Seq datasets from the International Cancer Genome Consortium (ICGC), GEO, and TCGA repositories. Authors found that *PUM1*, along with “novel” RGs - *CCSER2, SYMPK*, and *ANKRD17*, had the most stable mRNA level (Tilli et al., 2016). This agrees with previous qPCR analyses of RG expression stability in breast carcinomas (Ibusuki et al., 2013; Kilic et al., 2014).

*IPO8* (*importin 8*), which has 2 transcript isoforms and no pseudogenes, is the second in the cross-tissue stability list, but its mRNA level is much less stable than that of *PUM1* according to TCGA data. IPO8 mediates nuclear import of proteins with a classical nuclear localization signal. Previously, *IPO8* was found to be suitable for data normalization in endometrial (Ayakannu et al., 2015) and ovarian carcinomas (Kolkova et al., 2013), colon adenocarcinoma cell lines (Krzystek-Korpacka et al., 2016), non-small cell lung cancer (Soes et al., 2013), and other tissues and diseases: brain edema (Du et al., 2017), heart cavities (Molina et al., 2018), T cells, and neutrophils (Ledderose et al., 2011).

The *RPN1* gene (0 pseudogenes, 1 transcript isoform), which was previously suggested by us for normalization of qPCR data in LUAD, LUSC, KIRC, KIRP, and COAD (Krasnov et al., 2011; Fedorova et al., 2015), demonstrate stable expression in these cancer types as well as in PRAD, LIHC, and THCA.

The majority of the remaining genes from the set of 32 genes, even if they demonstrate stable mRNA level in certain cancer types, have many pseudogenes or high mutation rate (for example, *UBC* is above the 99th percentile in BRCA). The presence of pseudogenes is a weakness of such widely used RGs as *GAPDH* and *ACTB* (67 and 64, respectively) (Sun et al., 2012), or genes encoding ribosomal proteins, including *RPL13A* and *RPS17* (Tonner et al., 2012).

Next, we tried to find out novel reliable and convenient RGs suitable for most cancer types. As it was described above, for this purpose, we evaluated expression and mutation scorings for each examined cancer type, calculated pan-cancer scoring values given the “anti-scorings” for the number of transcript isoforms and pseudogenes, and selected the promising candidates taking into account information on functions of the genes and their involvement in carcinogenesis.

Along with *SFRS4* (number 13 in the top list of “universal” reference genes), three genes that participate in pre-mRNA splicing and processing pathways (*SF3A1, SF3B2*, and *SFRS3*) are present in the top 10 of promising pan-cancer RGs. The splicing machinery (namely spliceosome) is the largest molecular machine so far described. It is composed of five small nuclear ribonucleoproteins (snRNPs U1, U2, U4, U5, and U6) and more than 100 different polypeptides (Ghigna et al., 2008). Aberrant splicing in cancer provides a way to generate alternatively spliced transcripts encoding proteins with distinct functions (Ghigna et al., 2008). There are at least two ways resulting in splicing aberrations in cancer: mutations in the affected genes, e.g., in their splice sites (*cis*-effect), and altered expression and/or activity of the elements of splicing machinery (*trans*-effect). Some of the splicing factors are known to be deregulated in cancer, by means of mRNA level alterations, mutations or posttranslational modifications (Stickeler et al., 1999; Blaustein et al., 2005; Ghigna et al., 2008). On the other hand, some of the splicing factors are considered as potential RGs. This may be explained by the complexity of the splicing machinery and various roles of its elements (David and Manley, 2010).

*SF3A1* and *SF3B2* encode the subunits of splicing factors 3a and 3b. These two splicing factors together with 12S RNA unit form the U2 small nuclear ribonucleoproteins complex, which binds pre-mRNA upstream of the intron's branch site and may anchor the U2 snRNP to the pre-mRNA (Will et al., 2002). *SF3A1* is considered as a RG in sarcoma (Aggerholm-Pedersen et al., 2014), its expression was found to be stable in breast cancer (Maltseva et al., 2013), colorectal adenocarcinoma Caco-2 cells under exposure to food products (Vreeburg et al., 2011), white blood cells under treatment with growth hormone (Castigliego et al., 2010), bovine blastocysts produced by different methods (Luchsinger et al., 2014), bovine granulosa cells of dominant follicles during follicular growth and aging (Khan et al., 2016).

Considering the other splicing machinery gene, *SFRS4* (*serine and arginine rich splicing factor 4*), some authors earlier demonstrated that its mRNA level is stable in hepatocellular carcinoma (HCC) cell lines (Liu et al., 2017) and patients with alcoholic liver disease (Boujedidi et al., 2012). *SFRS4* remains stably expressed in hepatitis C virus-induced HCC, whereas *ACTB* and *GAPDH* are significantly deregulated (Waxman and Wurmbach, 2007).

CIAO1 (number 9 in the top list) is a key component of the cytosolic iron-sulfur protein assembly (CIA) complex. This is a multiprotein complex that mediates the incorporation of iron-sulfur cluster into extramitochondrial Fe/S proteins (provided by GeneCards; Stelzer et al., 2016). *CIAO1* was not previously described as a RG. Till now, there is only one article describing the possible role of the encoded protein in cancer development, namely interacting with the tumor suppressor protein WD40 (Johnstone et al., 1998). Besides this, there is almost no data on the association of this gene with cancer.

## Conclusions

To reveal reliable RGs for qPCR data normalization, a comprehensive analysis of TCGA data was performed. We took into account expression stability, average mRNA level, expression correlation with clinical and pathological characteristics, number of pseudogenes and transcript isoforms, mutation rate, GO terms, and mentions of a gene in titles/abstracts of articles from PubMed. The most reliable pairs of traditionally used RGs were suggested for each of 12 examined cancer types, as well as unsuitability of some frequently used RGs was shown. Pan-cancer analysis revealed promising RG candidates with stable and sufficiently high expression level and low mutation rate across 12 cancer types. Besides, these genes have only one known transcript isoform and no pseudogenes.

## Data Availability

All datasets generated for this study are included in the manuscript and/or the supplementary files.

## Author Contributions

GK, AK, NM, and AD conceived and designed the work. GK, AK, AS, VL, AB, NM, and AD performed data analysis. GK and AD wrote the manuscript. All authors agreed with the final version of the manuscript and all aspects of the work.

### Conflict of Interest Statement

The authors declare that the research was conducted in the absence of any commercial or financial relationships that could be construed as a potential conflict of interest.
